# Supplementary material for: Exploring strong and weak topological states on isostructural substitutions in TlBiSe2
Source: Sci Rep. 2022 Dec 20;12:21970. doi: 10.1038/s41598-022-26445-y (PMC9767939; doi:10.1038/s41598-022-26445-y)
Supplement: Supplementary file 1 — Supplementary Information. [file 41598_2022_26445_MOESM1_ESM.pdf]

Exploring Strong and Weak Topological States on Isostructural Substitutions in $TlBiSe_2$

Ankita Phutela,* Preeti Bhumla, Manjari Jain, and Saswata Bhattacharya*

Department of Physics, Indian Institute of Technology Delhi, New Delhi, India

E-mail: ankita@physics.iitd.ac.in[AP]; saswata@physics.iitd.ac.in[SB]

Phone: +91-11-2659 1359. Fax: +91-11-2658 2037

Supplementary Information

I. Band profile of $TlBiSe_2$ using PBE+SOC and G_0W_0@PBE+SOC ϵ_{xc} functionals.

II. Band inversion mechanism for $GaBiSe_2$ and $PbBiSe_2$.

III. Band structures of $SnBiSe_2$, $SbBiSe_2$, Bi_2Se_2, $TlSnSe_2$ and $PbSbSe_2$.

IV. Optimized lattice parameters of $SnBiSe_2$, $SbBiSe_2$, Bi_2Se_2, $TlSnSe_2$ and $PbSbSe_2$.

V. Phonon band structures of different configurations showing band inversion.

VI. Surface state band structures of $SnBiSe_2$, $SbBiSe_2$, Bi_2Se_2, $TlSnSe_2$ and $PbSbSe_2$.

VII. Band gap (in meV) of different materials using G_0W_0@HSE06+SOC ϵ_{xc} functional.

Band profile of $TlBiSe_2$ using PBE+SOC and G_0W_0@PBE+SOC ϵ_{xc} functionals.

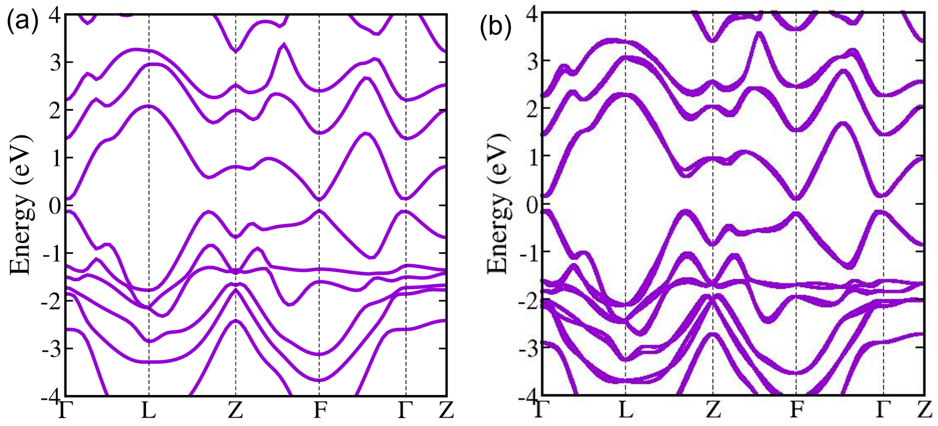

Figure S1: Band structure of $TlBiSe_2$ calculated using (a) PBE+SOC (b)G_0W_0@PBE+SOC.

Band inversion mechanism for $GaBiSe_2$ and $PbBiSe_2$.

Band inversion is an essential condition for topological insulators, in which the usual ordering of the conduction and valence band is inverted by spin-orbit coupling (SOC). For instance, the band inversion in $GaBiSe_2$ takes place between the p orbitals of Bi and Se. The p states of Bi lie above the fermi level, whereas p states of Se lie below the fermi level as seen in Figure S2(a). The inclusion of SOC brings band inversion at the Γ point where the Bi p states lies below the Se p states (see Figure S2(b)). This band ordering at Γ is opposite to that away from the zone center. In the case of $PbBiSe_2$, the switching in this band order takes place at two k-points, i.e., Γ and F. At these k-points, the band ordering becomes opposite on including SOC, i.e., the Se p orbitals become occupied and Bi p orbitals become unoccupied (see Figure S2 (c),(d)).

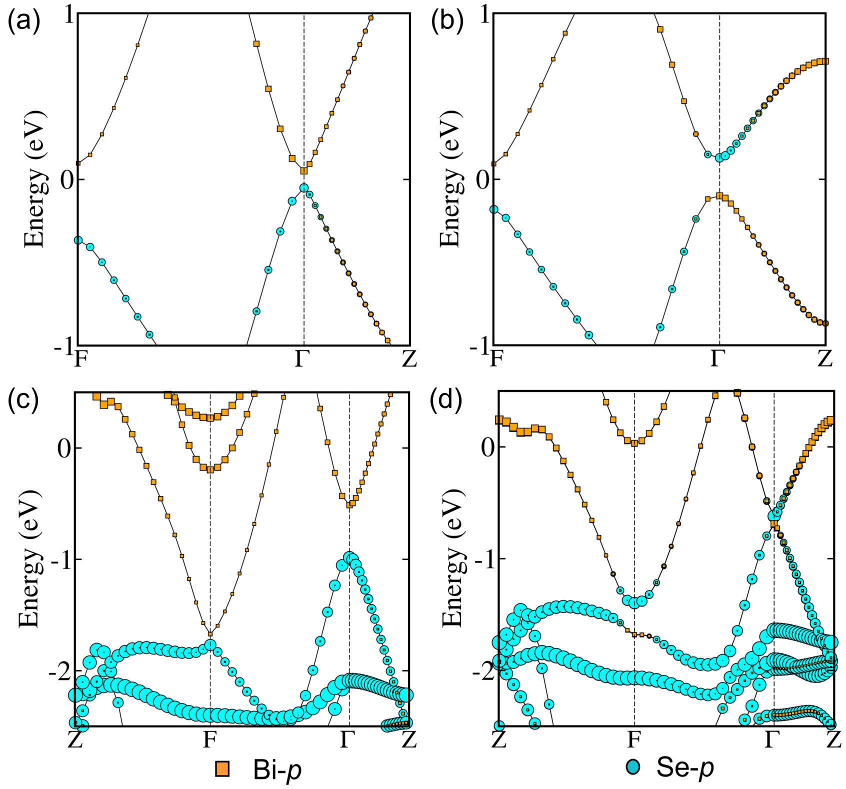

Figure S2: The band structure for $GaBiSe_2$ at Γ in (a) natural order, (b) inverted order and for $PbBiSe_2$ at F and Γ in (c) natural order and (d) inverted order, showing the band inversion.

Band structures of $SnBiSe_2$, $SbBiSe_2$, Bi_2Se_2, $TlSnSe_2$ and $PbSbSe_2$.

The band structure of $SnBiSe_2$ without and with SOC is shown in Figure S3(a) and S3(b), respectively. The band inversion occurs at two TRIMs i.e., Γ and F and it involves the *p* orbital of Bi and Se. Similar trend has been observed in $SbBiSe_2$, Bi_2Se_2, $TlSnSe_2$ and $PbSbSe_2$. In all of them, parity inversion is between the *p* orbital of Bi and Se at even number of TRIMs.

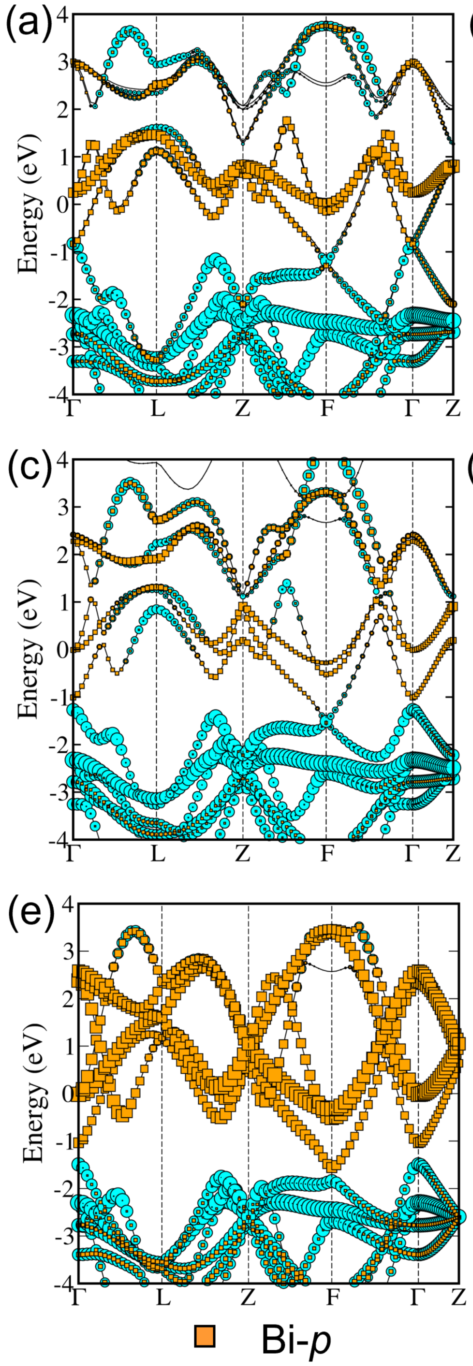

Figure S3: The band structures for $SnBiSe_2$, $SbBiSe_2$ and Bi_2Se_2 without SOC are shown in (a), (c), (e) and with SOC are shown in (b), (d), (f), respectively.

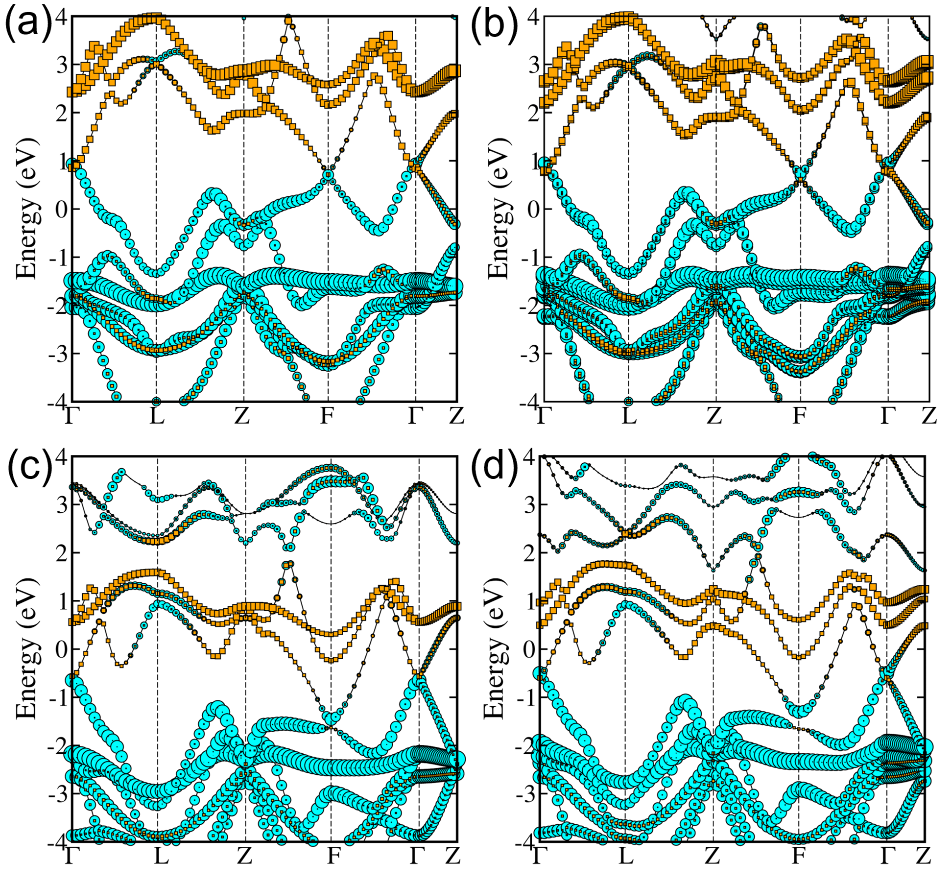

Figure S4: The band structures for $TlSnSe_2$ and $PbSbSe_2$ without SOC are shown in (a), (c) and with SOC are shown in (b), (d), respectively.

Optimized lattice parameters of $SnBiSe_2$, $SbBiSe_2$, Bi_2Se_2, $TlSnSe_2$ and $PbSbSe_2$.

Table S1: Optimized lattice parameters

Material	Lattice Parameters(Å)
$SnBiSe_2$	8.254
$SbBiSe_2$	8.059
Bi_2Se_2	8.163
$TlSnSe_2$	7.747
$PbSbSe_2$	8.166

Phonon band structures of different configurations showing band inversion. To analyze the dynamical stability, we have plotted the phonon band structures for $SnBiSe_2$, $SbBiSe_2$, Bi_2Se_2, $TlSnSe_2$ and $PbSbSe_2$. Figure S5 shows the phonon band structures calculated along the high-symmetry points of the BZ. Notably, the absence of negative frequencies in all of them confirm the dynamical stability of these materials.

Apart from the materials discussed in manuscript, $InBiSe_2$, $TlSbSe_2$, $TlHgSe_2$, $TlBiI_2$ and $GaHgSe_2$ also showed the band inversion. We have screened all of these materials based on their dynamical stability. As shown in Figure S6, none of these is dynamically stable, hence no further analysis is carried out on $InBiSe_2$, $TlSbSe_2$, $TlHgSe_2$, $TlBiI_2$ and $GaHgSe_2$. The other materials do not show parity inversion anywhere in the Brillouin Zone, therefore, they are characterized as trivial.

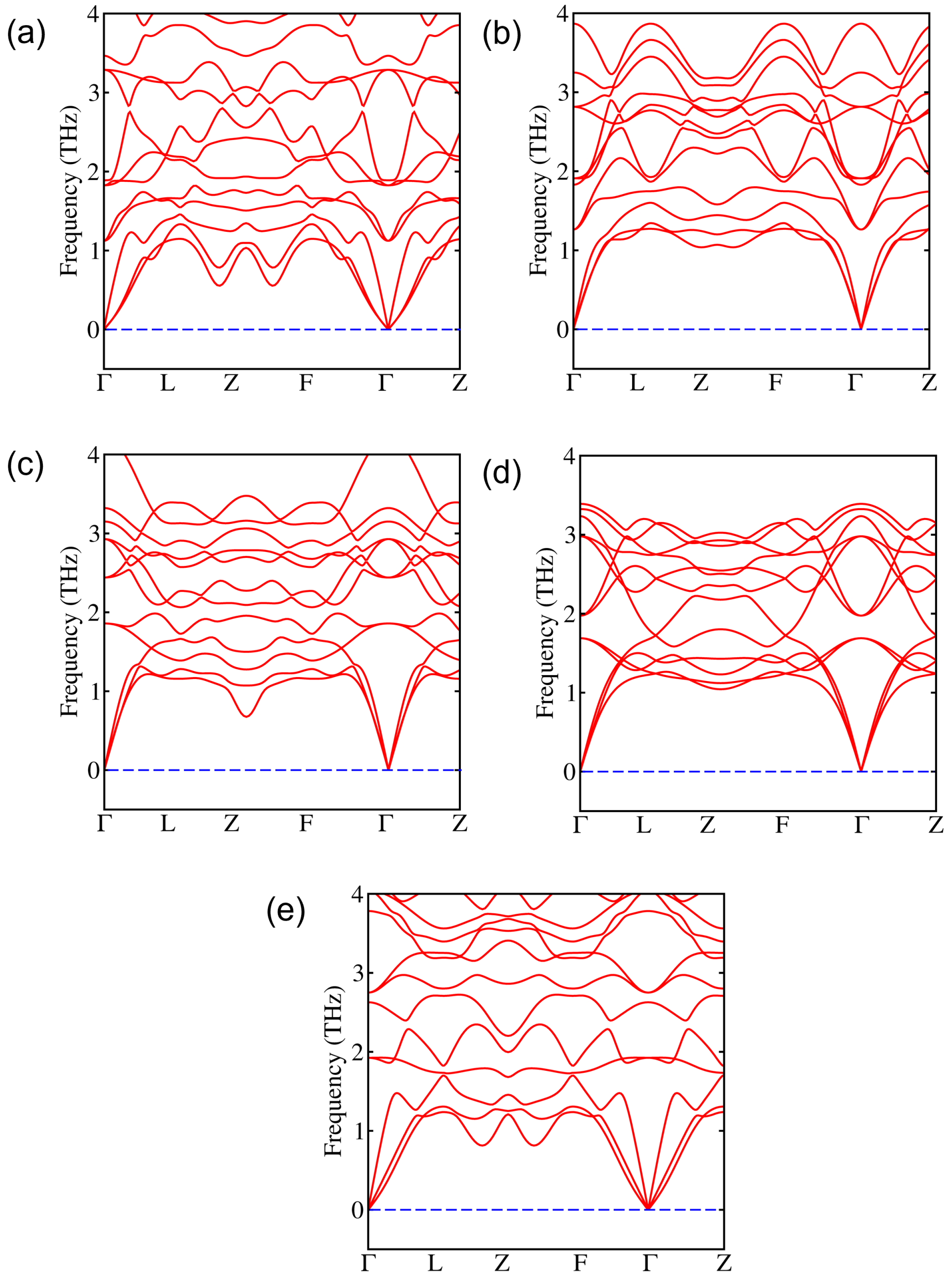

Figure S5: Phonon band structures of (a) $SnBiSe_2$, (b) $SbBiSe_2$, (c) Bi_2Se_2, (d) $TlSnSe_2$ and (e) $PbSbSe_2$.

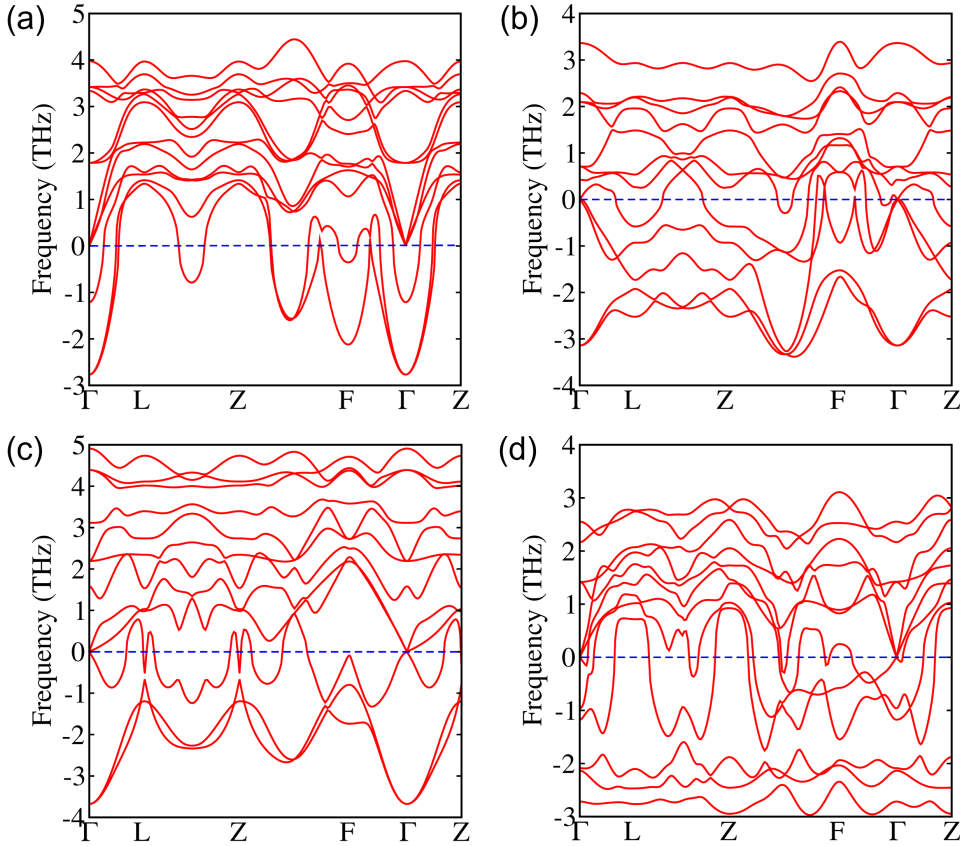

Figure S6: Phonon band structures of (a) $InBiSe_2$, (b) $TlHgSe_2$, (c) $TlBiI_2$ and (d) $GaHgSe_2$.

Surface state band structures of $SnBiSe_2$, $SbBiSe_2$, Bi_2Se_2, $TlSnSe_2$ and $PbSbSe_2$.

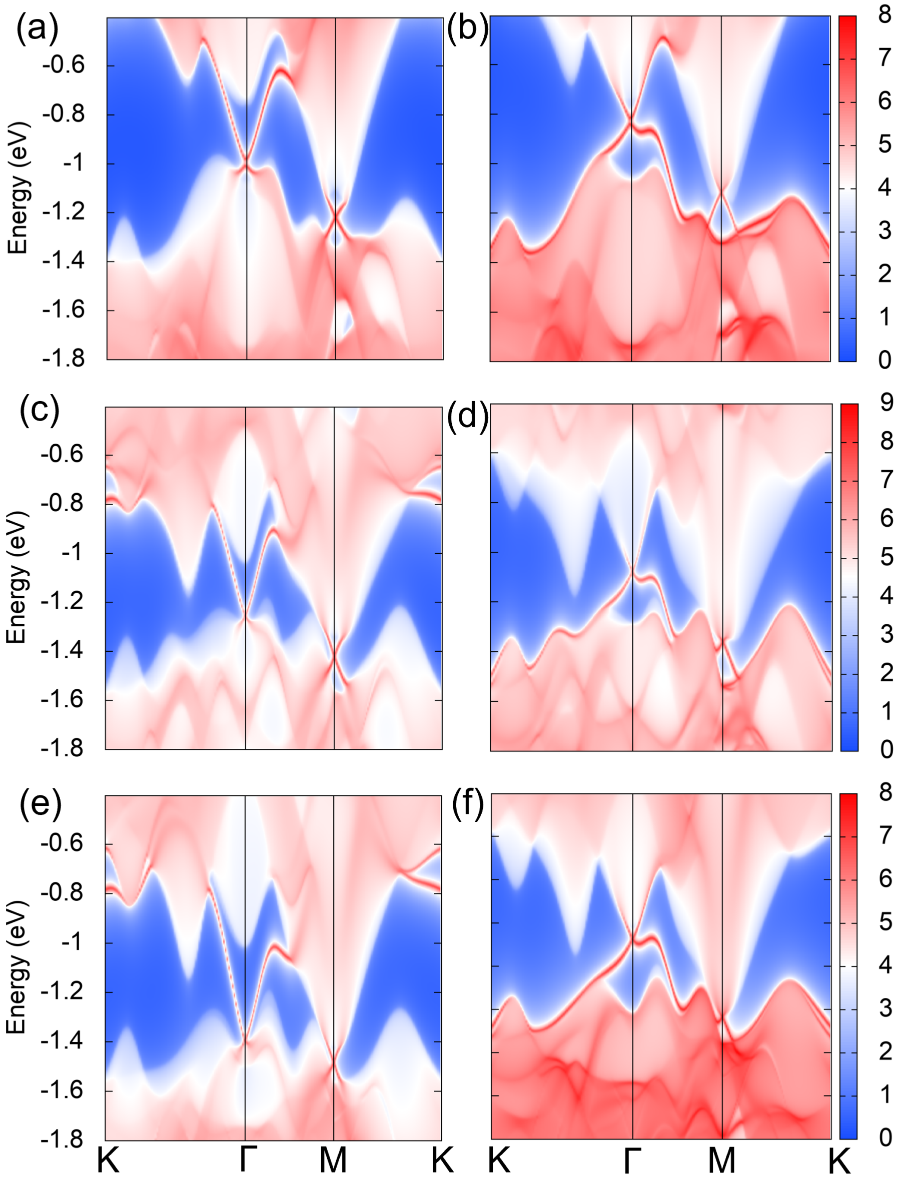

Figure S7: Surface states for the left surface of $SnBiSe_2$, $SbBiSe_2$ and Bi_2Se_2 are shown in (a), (c), (e) and for the right surface are shown in (b), (d), (f), respectively.

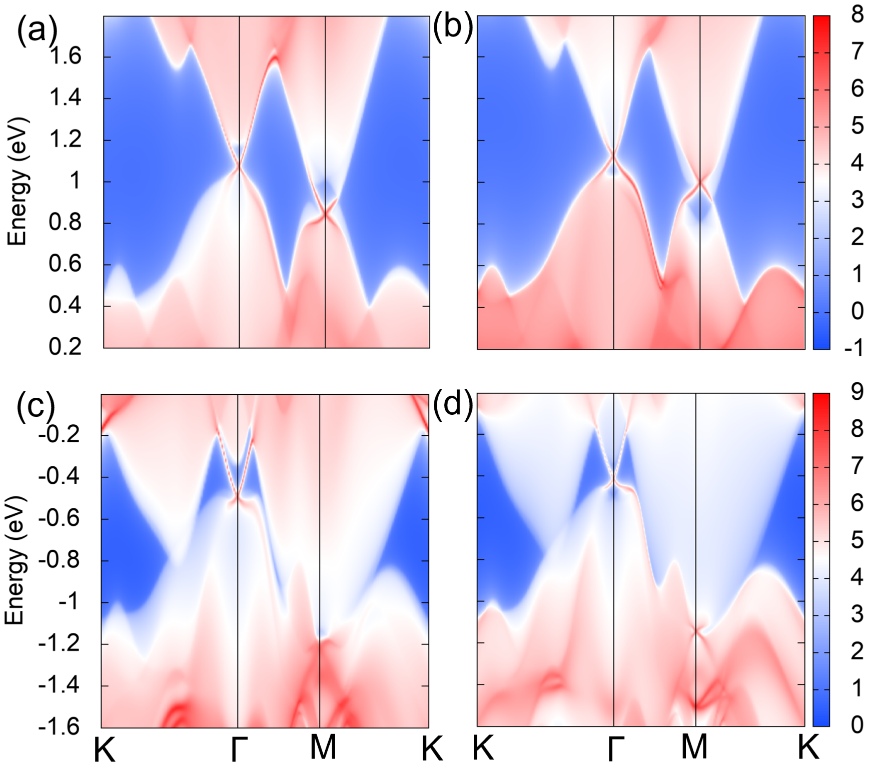

Figure S8: Surface states for the left surface of $TlSnSe_2$ and $PbSbSe_2$ are shown in (a), (c) and for the right surface are shown in (b), (d), respectively.

Band gap (in meV) of different materials using G_0W_0@HSE06+SOC ϵ_{xc} functional.

Table S2: Band gap using G_0W_0@HSE06+SOC ϵ_{xc} functional.

Material	Indirect Band Gap (meV)
**$GaBiSe_2$**	135
**$PbBiSe_2$**	62
**$SnBiSe_2$**	11
**$SbBiSe_2$**	85
**Bi_2Se_2**	122
**$TlSnSe_2$**	43
**$PbSbSe_2$**	166
